# Growth pattern in Ethiopian infants – the impact of exposure to maternal HIV infection in relation to socio-economic factors

**DOI:** 10.1080/16549716.2017.1296726

**Published:** 2017-05-04

**Authors:** John König Walles, Taye Tolera Balcha, Niclas Winqvist, Per Björkman

**Affiliations:** ^a^ Department of Translational Medicine, Section for Infectious Diseases, Lund University, Malmö, Sweden; ^b^ Department of Infectious Diseases, Central Hospital, Kristianstad, Sweden; ^c^ Armauer Hansen Research Institute, Addis Ababa, Ethiopia; ^d^ Skåne Regional Office for Infectious Disease Control and Prevention, Malmö, Sweden

**Keywords:** HIV-EU, infant malnutrition, PMTCT, Ethiopia

## Abstract

**Background**: Infants exposed to maternal HIV infection who remain HIV-uninfected (HIV-exposed/uninfected; HIV-EU) may be at increased risk of growth retardation, which could be due both to directly HIV-related effects and to socio-economic factors overrepresented among HIV-positive women.

**Objective**: To investigate growth development at 9–12 months of age in HIV-EU infants participating in prevention of mother-to-child transmission (PMTCT) care compared to HIV unexposed (HIV-U) infants in relation to socio-economic conditions.

**Methods**: Anthropometric and socio-economic data were collected retrospectively from PMTCT registers (for HIV-EU infants), with HIV-U controls recruited at measles vaccination at public health facilities in Ethiopia. Growth was compared with regard to HIV exposure and socio-economic variables in multivariate regression analysis.

**Results**: The following growth measurements were found for 302 HIV-EU and 358 HIV-U infants at 9–12 months of age, respectively: mean weight-for-age z-score (WAZ) 0.04 and −0.21, *p* < 0.001 (proportion underweight 5.7% and 6.7%, *p* = 0.60); median length-for-age z-score (LAZ) −0.92 and −0.91, *p* = 0.53 (proportion stunted 25.1% and 20.5%, *p* = 0.17). In multivariate analysis, lower WAZ was associated with male sex (*p* = 0.021), lower maternal education (*p* < 0.001), presence of siblings (*p* < 0.01) and HIV-U (*p* < 0.01). Underweight was associated with male sex (*p* = 0.017) and absence of maternal education (*p* = 0.019). Lower LAZ was associated with male sex (*p* < 0.001), presence of siblings (*p* < 0.001) and poor maternal education (*p* < 0.01), while stunting was associated with male sex (*p* < 0.001), presence of siblings (*p* < 0.001), few rooms in the home (*p* < 0.01), access to running water (*p* = 0.026) and low level of maternal education (*p* = 0.014).

**Conclusions**: At 9–12 months of age, HIV-EU infants had non-inferior growth and higher mean WAZ than HIV-U controls. Poor growth development was associated with socio-economic factors. This suggests health benefits from PMTCT participation for infant growth. Similar interventions could be considered for Ethiopian infants, irrespective of HIV exposure, with a particular focus on children with poor socio-economic status.

## Background

Malnutrition is a major contributor to infant morbidity and mortality in sub-Saharan Africa, caused by insufficient intake of calories as well as increased energy expenditure from infections and other types of stress [1]. Acute malnutrition is characterized by rapid onset of weight loss, is associated with high mortality and is usually reversible with nutritional support. On the other hand, chronic malnutrition has long-term consequences such as poor length gain and inferior neuro-cognitive development [1–3]. Anthropometric measurements are used for determination of nutritional status, both for individual assessment and for population surveys. Acute malnutrition is defined as weight-for-age Z-score (WAZ) more than 2 standard deviations below the mean (WAZ < –2, underweight) according to World Health Organization (WHO) growth standards [4]. Chronic malnutrition is diagnosed using length-for-age as the indicator, and stunting refers to length-for-age Z-score (LAZ) < –2 [3,4]. Infant malnutrition is most common in low-income countries, particularly in sub-Saharan Africa. In 2010, 18% of children under five were underweight and 26% were stunted [5].

A range of factors related to poverty, such as low parental education, short sibling interval, poor access to health care, episodes of diarrhoea, poor food diversity and not practising exclusive breastfeeding, have been associated with acute and chronic malnutrition [6–8]. In parallel with malnutrition, sub-Saharan Africa is home to the majority of vertically HIV-infected children globally. Infant HIV infection is associated with high rates of morbidity and mortality [9], as well as growth retardation. With increasing access to prevention of mother-to-child transmission (PMTCT) interventions and antiretroviral therapy (ART) for pregnant women, the number of HIV-infected children is expected to decrease. However, with improving health of HIV-positive women, more children who are exposed to maternal HIV infection during early life will remain HIV-uninfected (HIV exposed/uninfected; HIV-EU) [10]. Despite not acquiring HIV infection, several adverse health outcomes have been reported in HIV-EU children, including elevated mortality [11,12] and morbidity, whereas the effect on growth pattern varies between studies [10,13,14]. The mechanisms, however, are not fully understood.

An important component of PMTCT programs is follow-up of children born to HIV-positive mothers until HIV serostatus can be definitely determined. Early infant diagnosis of HIV infection allows for early initiation of treatment, but apart from repeated HIV testing, these infants also receive targeted interventions, including antiretroviral and cotrimoxazole prophylaxis, and usually also nutritional support in case of suspected or confirmed malnutrition.

The growth deviations observed in HIV-EU children may be directly caused by HIV exposure in utero and during breastfeeding, but could also be due to co-existing factors, in particular poor socio-economic status [15] and lack of maternal education [16]. The heterogeneity in reported findings on the impact on growth in HIV-EU may be explained by how control groups have been selected, and the distribution of co-existent factors that may be associated with growth disturbances. Study populations from different time periods and geographical regions also differ regarding access to ART and PMTCT care.

Ethiopia is home to a large population of HIV-positive women of reproductive age, 1.9% in 2011 [17]. Since 2005, access to ART and PMTCT has increased in the country; in 2014 65% of HIV-infected pregnant women were estimated to participate in PMTCT programs [18]. Child malnutrition remains an important public health problem; in 2011, 31.6% were stunted and 29.1% were underweight among 9–11-month-old Ethiopian infants [17]. To our knowledge, the relationship between infant growth, HIV exposure and socio-economic factors has not previously been investigated in an Ethiopian setting. We hypothesized that the increased rate of malnutrition in HIV-EU observed in some studies may be influenced by confounding socio-economic factors.

In this study, we aimed to determine factors associated with growth pattern in Ethiopian infants 9–12 months of age, including HIV exposure as one potential risk factor as well as a range of socio-economic variables.

## Methods

### Study setting

This cross-sectional study was performed in four health centres (Adama, Geda, Mojo and Wolenchiti) and the Adama Regional Hospital in the Oromia region, Central Ethiopia (representing all public health facilities providing HIV care in the uptake area populated by 600,000 inhabitants). The adult HIV prevalence here is higher than in other parts of Ethiopia (9% of pregnant women in 2005 [19]). While antenatal care (ANC) and PMTCT care are practised at both the hospital and the four health centres, immunization is only provided at health centres. At ANC registration and at delivery women are offered free HIV testing and if HIV infection is confirmed the woman is referred for HIV care [20]. At HIV care enrolment, extensive structured data on socio-economic and demographic factors are recorded.

During the period when mothers whose infants were included in this study were pregnant, national recommendations for PMTCT practice were changed. For the whole study period, triple drug antiretroviral was initiated at diagnosis and continued lifelong for pregnant women with advanced disease with respect to C.D.4 counts and WHO clinical stage. For women with less severe disease, initial recommendations of antiretroviral prophylaxis from 14th gestational week and until one week postpartum were replaced from 2010 by recommendations for triple drug ART from 14th gestational week until one week after cessation of breastfeeding. For infants, single-dose nevirapine at delivery was used for prophylaxis [20].

HIV-exposed infants are enrolled for PMTCT at 6 weeks of age. Basic demographic data are recorded, together with anthropometric measurements and signs of infection or malnutrition. The dried bloodspot (DBS) test for HIV DNA is done at this time. Irrespective of HIV DNA result, cotrimoxazole prophylaxis is started and the infant is followed monthly until 6 months of age, and subsequently every three months up to the age of 18 months when HIV status is finally determined using the antibody detection test. Advice on infant health care is given continuously, advocating exclusive breastfeeding for 6 months followed by rapid weaning if deemed safe and feasible. If criteria for malnutrition (any anthropometric measurement below the third percentile, pitting oedema or other clinical signs) are met, nutritional support is provided free of charge [20].

### HIV-EU infants

PMTCT registers at the study sites were used to identify HIV-exposed infants enrolled in PMTCT with linkage to medical records containing basic demographic data including caretaker’s name and medical record number, in addition to anthropometric measurements and other infant health-related data for each follow-up visit. Data on growth, sex, feeding practice and HIV status were collected from the first visit during the period between 270 and 365 days of age. For each HIV-EU infant, the corresponding maternal medical record was traced. From these records, socio-economic and demographic data were collected and registered in a database linked to infant data. This information is recorded from all HIV-positive women at registration using structured forms. WAZ and LAZ were determined for each child according to growth standards [4] using software provided by the WHO [21]. Underweight and stunting were defined and determined similarly. All HIV-exposed infants in the PMTCT registers of the five study sites from introduction of the PMTCT registers until 270 days before data collection (July 2007 to January 2013) were eligible for inclusion. Children with confirmed HIV infection at any time, as well as cases for which infant and/or maternal records could not be retrieved, were excluded.

### HIV-U infants

HIV-U controls were consecutively recruited from immunization clinics at the health centre study sites during a period of 7 weeks starting in September 2013. Infant–mother pairs coming for measles vaccination from 9 months of age were eligible for inclusion. Infants were measured and weighed by trained personnel, and the mothers were interviewed using a questionnaire covering the same information as that obtained during maternal HIV care and PMTCT follow-up. Children born to mothers with known HIV infection were excluded. Mechanical scales were used for weight measurements. Children with signs of malnutrition were managed according to Ethiopian national guidelines.

### Statistical analysis

For analysis of factors associated with growth patterns, data from HIV-EU and HIV-U infants were merged, and HIV exposure was included as one variable. In addition, the following variables were included in this analysis. Demographic: number of living and dead siblings, vital status of parents; socio-economic: number of persons sharing the home, number of rooms in the home, electricity and access to running water in the home, rural or urban area of residency, maternal marital status, highest level of completed education and employment status; health and reproductive: paternal HIV status, mode of delivery, maternal age at time of her first delivery and delivery of the study participant, and feeding practice during the first 6 months and at time of visit. Aiming to be able to identify a 50% increase in malnutrition among the HIV-EU infants compared to 30% in the general population, a sample size of 190 infants in each group was necessary for a power of 0.90.

The relative frequencies and odds ratios (OR) of socio-economic and other infant characteristics with respect to HIV exposure were calculated. Univariate analysis was performed for individual correlations between the continuous outcome variables – WAZ and LAZ – and the socio-economic and demographic factors listed in Table 1. Multivariate regression analysis and backward selection were performed until separate models for WAZ and LAZ respectively only contained factors with statistically significant impact on the outcome variables that remained. For the categorical outcome variables underweight and stunted, analysis was performed similarly but using logistic regression with backward selection, and statistically significant impact on OR for inclusion in the models. All statistical analysis was performed using the IBM SPSS Statistics 22 software.

**Table 1.** Infant and family characteristics for HIV unexposed and exposed/uninfected.

	HIV unexposed (HIV-U)		HIV exposed/uninfected (HIV-EU)		HIV-EU compared to HIV-U^1^
Characteristic	N	%	N	%	OR (95% CI)^2^
*Gender*					
Female	165	46	154	51	Ref
Male	193	54	148	49	0.82 (0.60–1.12) NS
*Feeding at 9–12 months*					
Breastfed	321	91	230	76	Ref
Weaned	33	9	72	24	3.05 (1.95–4.75)***
*Number of siblings*					
> 1	92	29	90	31	1.10 (0.78–1.56) NS
1	136	43	102	35	0.72 (0.52–1.00) NS
0	88	28	97	34	1.31 (0.93–1.85) NS
*Maternal age at first birth (years)*					
< 20	99	28	99	37	1.50 (1.07–2.11)*
20–29	239	68	151	56	0.62 (0.44–0.85)**
> 29	16	5	19	7	1.61 (0.81–3.18) NS
Maternal age at current birth (years)					
< 20	23	7	5	2	0.21 (0.01–0.58)**
20–29	224	73	177	61	0.59 (0.41–0.84)**
> 29	60	20	106	37	2.40 (1.66–3.47)***
Rooms in house					
> 2	107	31	41	16	0.42 (0.28–0.62)***
2	100	29	84	32	1.17 (0.82–1.65) NS
1	143	41	139	53	1.61 (1.16–2.22)**
Electricity in house					
Yes	308	87	183	69	Ref
No	48	13	83	31	2.91 (1.95–4.34)***
Running water in house					
Yes	264	74	138	52	Ref
No	91	26	128	48	2.69 (1.91–3.78)***
Persons living in the home					
< 3	9	3	79	31	16.2 (7.96–33.1)***
3–5	295	87	143	55	0.19 (0.13–0.28)***
> 5	36	11	36	14	1.37 (0.84–2.24) NS
Maternal education					
Tertiary	73	21	15	5	0.21 (0.12–0.37)***
Secondary	120	34	75	26	0.67 (0.48–0.95)*
Primary	120	34	125	43	1.55 (1.12–2.14)*
None	42	12	78	27	2.70 (1.79–4.09)***
Working					
Yes	98	28	78	29	Ref
No	250	72	195	71	0.98 (0.69–1.39) NS
Currently married					
Yes	337	96	203	69	Ref
No	14	4	91	31	10.8 (5.99–19.4)***

Notes: ^1^Individual associations for HIV-EU compared to HIV-U. ^2^*p*-values are calculated from Pearson’s chi-square test. *p < 0.05, **p < 0.01, ***p < 0.001. NS: non-significant.

## Results

### Study participant characteristics

**Figure 1.**
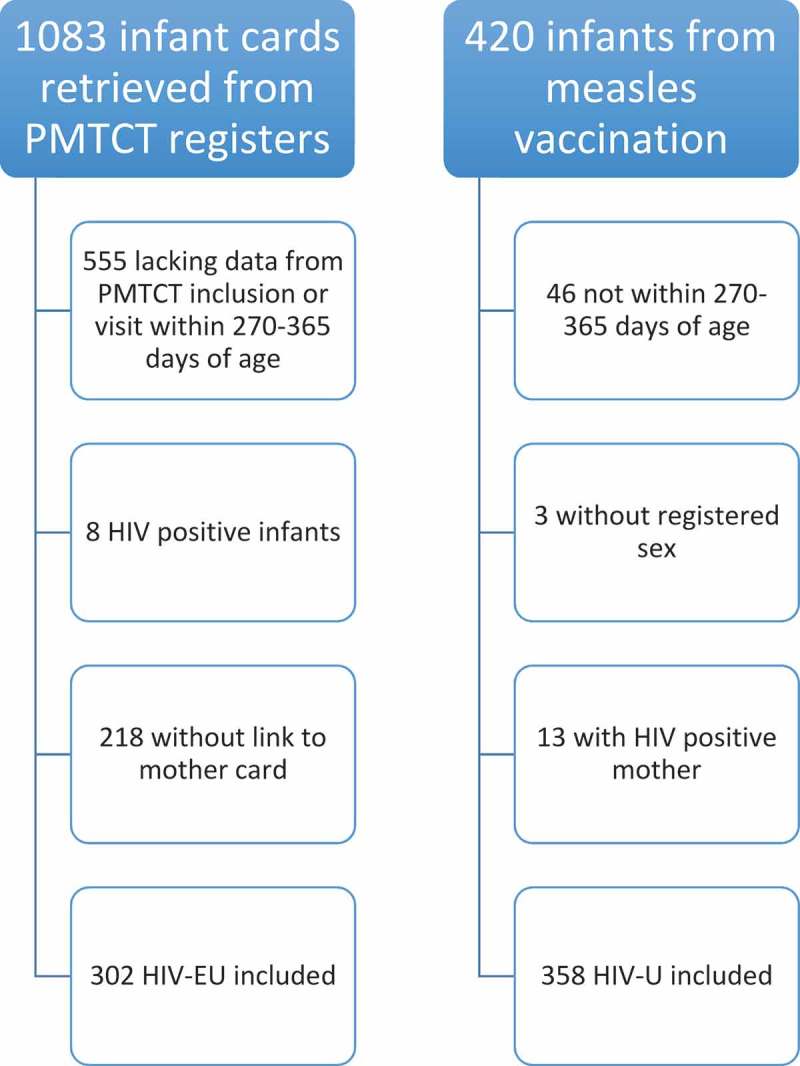
Exclusion process for HIV exposed uninfected (HIV-EU) and HIV unexposed (HIV-U) infants.

Out of 1083 infants identified in the PMTCT registers, 555 were not included because of lacking data from enrolment or a visit within 270–365 days of age, 8 infants were excluded because of confirmed HIV infection and 218 were excluded due to lacking data from the mothers’ medical records. Three hundred and two HIV-EU remained for inclusion (Figure 1). Of the 420 infants recruited from the immunization clinics, 46 were excluded since their age was not within 270–365 days. In 3 cases data on the infant sex were missing, and 13 subjects were excluded since their mothers were HIV-positive. Three hundred and fifty-eight HIV-U were included (Figure 1). Some factors were rarely specified in the questionnaires or ART intake forms respectively, and were therefore removed from further analysis. These were paternal vital and HIV status, number of dead siblings, mode of delivery, feeding practice until 6 months of age and rural vs urban residency.

### Socio-economic and demographic conditions and HIV exposure

Compared to HIV-negative mothers, HIV-infected mothers had a higher age diversity at first delivery but were older at the delivery of the study participant. Several poverty indicators (lack of electricity, lower number of rooms in the house, unavailability of running water) were also more common in this group (Table 1), as well as lower educational level. In addition, a lower proportion of HIV-positive women were married. Weaning before 9–12 months of age was also more common in HIV-EU infants but gender distribution and number of siblings were similar (Table 1).

### Growth parameters at 9–12 months of age

Details on growth parameters are shown in Table 2. At 9–12 months of age, mean WAZ was −0.099 and 6.3% (41 of 656) were underweight; mean LAZ was −0.91 and 22.6% (143 of 634) were stunted (Table 2). In HIV-EU, WAZ was significantly higher (0.04 compared to −0.21 among HIV-U, *p* < 0.01) while 5.7% (17 of 298) of HIV-EU were underweight compared to 6.7% (24 of 358) of HIV-U (*p* = 0.60). HIV-EU had equal LAZ (−0.92 compared to −0.91 for HIV-U, *p* = 0.53). The proportion of stunted infants was slightly higher among HIV-EU (25.1% vs 20.5% among HIV-U); however, this difference did not reach statistical significance (*p* = 0.17).

**Table 2.** Growth outcomes with regard to HIV exposure.

	HIV unexposed (HIV-U)		HIV exposed/uninfected (HIV-EU)		Total		
	Mean	SD/%	Mean	SD/%	Mean	SD/%	*p*
Weight-for-age Z-score (WAZ)	−0.21	1.16	0.04	1.26	−0.099	1.21	0.009^1^
Underweight	24/358	6.7	17/298	5.7	41/656	6.3	0.60^2^
Length-for-age Z-score (LAZ)	−0.91	1.59	−0.92	1.66	−0.91	1.62	0.94^1^
Stunted	71/347	20.5	72/287	25.1	143/634	22.6	0.17^2^

Notes: For Z-scores, means and standard deviations are presented. For proportions of underweight and stunted, numbers and proportions are presented. ^1^Two-sided *t*-tests are used for difference in means for WAZ and LAZ. ^2^Pearson’s chi-square tests for two independent proportions are used for underweight and stunted.

### Factors associated with WAZ and underweight at 9–12 months of age

Lower WAZ was associated with being HIV unexposed (adjusted difference [AD] −0.33; 95% confidence interval [CI] −0.52, −0.13; *p* < 0.01), male sex (AD −0.22; 95% CI −0.41, −0.03; *p* = 0.021), low level of maternal education (AD −0.65; 95% CI −1.01, −0.30; *p* < 0.001 for no education; and AD −0.49; 95% CI −0.81, −0.18; *p* < 0.01 for primary education; both compared to tertiary education), and two or more siblings (AD −0.33; 95% CI −0.57, −0.08; *p* < 0.01, compared to none) (Table 3). Increased risk for underweight was associated with no maternal education (adjusted odds ratio [AOR] 11.66; 95% CI 1.50–90.7; *p* = 0.019) compared with tertiary education, and male sex (AOR 2.34; 95% CI 1.17–4.69; *p* = 0.017) (Table 3).

**Table 3.** Weight development by HIV exposure, infant and family characteristics.

	Weight-for-age Z-score (WAZ)			Adj. difference in WAZ^1^	Underweight		AOR for underweight^2^
Characteristic	n	Mean	SD	B (95% CI)	n/N	%	AOR (95% CI)
*HIV exposure*							
Unexposed	358	−0.21	1.16	Ref	24/358	6.7	
Exposed/uninfected	298	0.04	1.26	0.33 (0.13–0.52)**	17/298	5.7	
*Gender*							
Female	315	0.01	1.17	Ref	12/315	3.8	Ref
Male	341	−0.20	1.25	−0.22 (−0.41, −0.03)*	29/341	8.5	2.34 (1.17–4.69)*
*Feeding practice at 9–12 months*							
Breastfed	549	−0.14	1.20		35/549	6.4	
Weaned	103	0.16	1.28		6/103	5.8	
Siblings							
0	185	0.09	1.18	Ref	10/185	5.4	
1	236	−0.06	1.16	−0.11 (−0.34–0.12) NS	13/236	5.5	
> 1	180	−0.31	1.22	−0.33 (−0.57, −0.08)**	14/180	7.8	
Maternal age at first birth (years)							
< 20	197	−0.29	1.18		16/197	8.1	
20–29	387	−0.06	1.19		21/387	5.4	
> 29	35	0.09	1.51		3/35	8.6	
Maternal age at current birth (years)							
< 20	28	−0.20	1.12		1/28	3.6	
20–29	400	−0.06	1.21		24/400	6	
> 29	165	−0.14	1.20		10/165	6.1	
Rooms in the home							
> 2	146	0.12	1.21		7/146	4.8	
2	184	−0.02	1.23		9/184	4.9	
1	280	−0.21	1.19		22/280	7.9	
Electricity							
Yes	489	−0.05	1.21		29/489	5.9	
No	129	−0.17	1.26		10/129	7.8	
Running water							
Yes	400	−0.08	1.23		25/400	6.3	
No	217	−0.07	1.21		14/217	6.5	
Persons living in the home							
> 5	71	−0.15	1.18		4/71	5.6	
3–5	435	−0.13	1.17		29/435	6.7	
< 3	88	0.13	1.39		4/88	4.5	
Maternal education							
Tertiary	87	0.24	1.22	Ref	1/87	1.1	Ref
Secondary	194	−0.04	1.20	−0.25 (−0.57–0.07) NS	13/194	6.7	6.27 (0.81–48.8) NS
Primary	244	−0.17	1.16	−0.49 (−0.81, −0.18)**	13/244	5.3	4.90 (0.63–38.1) NS
None	119	−0.32	1.30	−0.65 (−1.01, −0.30)***	14/119	11.8	11.7 (1.50–90.7)*
Working							
Yes	175	0.01	1.25		14/175	8	
No	442	−0.13	1.20		25/442	5.7	
Married							
Yes	536	−0.10	1.24		35/536	6.5	
No	105	−0.03	1.13		6/105	5.7	

Notes: ^1^Adjusted difference in WAZ in a multivariate regression analysis including HIV-EU, siblings, rooms and maternal education. ^2^Adjusted odds ratio for underweight in a logistic regression analysis including sex and maternal education. *p < 0.05, **p < 0.01, ***p < 0.001. NS: non-significant.

### Factors associated with LAZ and stunting at 9–12 months of age

Lower LAZ was associated with having siblings (AD −0.61; 95% CI −0.92, −0.30; *p* < 0.001 for one sibling; and AD −0.51; 95% CI −0.84, −0.18; *p* < 0.01 for two or more siblings as compared to absence of siblings), male sex (AD −0.47; 95% CI −0.72, −0.21]; *p* < 0.001), and no maternal education (AD −0.72; 95% CI −1.20, −0.24; *p* < 0.01, compared to tertiary education) (Table 4). The risk of being stunted was associated with male sex (AOR 2.20; 95% CI 1.42–3.42; *p* < 0.001), having siblings (AOR 2.98; 95% CI 1.69–5.25; *p* < 0.001 for one sibling; and AOR 2.20; 95% CI 1.18–4.12; *p* = 0.014, for two or more compared to absence of siblings), having only one room (AOR 2.34; 95% CI 1.27–4.67; *p* < 0.01) compared to three or more, and no maternal education (AOR 3.22; 95% CI 1.27–8.22; *p* = 0.014, compared to tertiary education) (Table 4). Unavailability of running water was associated with reduced risk for stunting (AOR 0.57; 95% CI 0.35–0.94; *p* = 0.026) (Table 4).

**Table 4.** Longitudinal growth by HIV exposure, infant and family characteristics.

	Length-for-age Z-score (LAZ)			Adj. difference in LAZ^1^	Stunted		AOR for stunting^2^
Characteristic	n	Mean	SD	B (95% CI)	n/N	%	AOR (95% CI)
*HIV exposure*							
Unexposed	347	−0.91	1.59		71/347	20.5	
Exposed/uninfected	287	−0.92	1.66		72/287	25.1	
*Gender*							
Female	305	−0.71	1.45	Ref	52/305	17	Ref
Male	329	−1.10	1.75	−0.47 (−0.72, −0.21)***	91/329	27.7	2.20 (1.42–3.42)***
Feeding at 9–12 months							
Breastfed	530	−0.98	1.59		121/530	22.8	
Weaned	100	−0.52	1.72		20/100	20	
Siblings							
0	182	−0.52	1.47	Ref	26/182	14.3	Ref
1	228	−1.15	1.60	−0.61 (−0.92, −0.30)***	67/228	29.4	2.98 (1.69–5.25)***
> 1	172	−1.11	1.69	−0.51 (−0.84, −0.18)**	41/172	23.8	2.20 (1.18–4.12)*
Maternal age at first birth (years)							
< 20	190	−1.09	1.56		50/190	26.3	
20–29	378	−0.92	1.63		83/378	22	
> 29	33	−0.29	1.65		5/33	15.2	
Maternal age at current birth (years)							
< 20	26	−0.94	1.30		5/26	19.2	
20–29	388	−0.98	1.70		99/388	25.5	
> 29	159	−0.87	1.56		31/159	19.5	
Rooms in the home							
> 2	138	−0.53	1.51		19/138	13.8	Ref
2	181	−0.98	1.60		37/181	20.4	1.85 (0.94–3.77) NS
1	276	−1.06	1.63		75/276	27.2	2.34 (1.27–4.67)**
Electricity							
Yes	478	−0.85	1.62		97/478	20.3	
No	125	−1.12	1.53		35/125	28	
Running water							
Yes	391	−0.96	1.68		91/391	23.3	Ref
No	211	−0.81	1.46		41/211	19.4	0.57 (0.35–0.94)*
Persons living in the home							
> 5	70	−0.90	1.34		12/70	17.1	
3–5	424	−0.94	1.61		96/424	22.6	
< 3	86	−0.80	1.69		19/86	22.1	
Maternal education							
Tertiary	86	−0.49	1.60	Ref	9/86	10.5	Ref
Secondary	189	−0.87	1.51	−0.22 (−0.65–0.21) NS	40/189	21.2	1.68 (0.71–3.95) NS
Primary	228	−0.86	1.57	−0.33 (−0.75–0.10) NS	51/228	22.4	2.1 (0.90–4.90) NS
None	119	−1.38	1.82	−0.72 (−1.20, −0.24)**	38/119	31.9	3.22 (1.27–8.22)*
Working							
Yes	173	−0.71	1.66		30/172	17.3	
No	430	−1.01	1.59		104/430	24.2	
Currently married							
Yes	518	−0.89	1.62		116/518	22.4	
No	104	−1.03	1.63		24/104	23.1	

Notes: ^1^Adjusted difference in LAZ in a multivariate regression including sex, siblings and maternal education. ^2^Adjusted odds ratio for stunting in a logistic regression analysis for sex, siblings, rooms and running water in the home and maternal education. *p < 0.05, **p < 0.01, ***p < 0.001. NS: non-significant.

## Discussion

The potential impact of maternal HIV exposure during early life on growth pattern in infants who remain HIV-uninfected is controversial. In order to investigate factors that may be involved in growth retardation observed in HIV-EU children in sub-Saharan Africa, we analysed growth parameters at 9–12 months of age in infants from the same uptake area in Ethiopia with regard to exposure to maternal HIV infection and socio-economic and demographic factors known to be associated with infant growth. Despite poorer socio-economic conditions among HIV-EU infants, rates of underweight and stunting were similar to those found in HIV-U subjects. Furthermore, mean LAZ scores were not significantly different in these populations, and HIV-EU was associated with higher WAZ scores. Inferior growth was associated with lack of maternal education, presence of older siblings and male gender – risk factors for growth retardation that have previously been found in general populations and HIV-EU in low-income countries [16,22–24].

As a consequence of improved access to ART and reduced rates of MTCT in sub-Saharan Africa the number of HIV-EU is growing. Child malnutrition and growth impairment remain widespread in this region. Families affected by HIV may be more vulnerable to food insecurity [25,26], and interactions between infant HIV exposure and poor socio-economic conditions may obscure true causative relationships. Infant malnutrition and growth retardation are multifactorial, and lead to adverse outcomes in both the short and the long terms, including high acute mortality, susceptibility to various infections, poor cognitive development and even foetal growth retardation and other obstetric complications in the next generation [3,27,28]. Girma and co-workers identified poverty with lack of proper food and sanitation, lack of maternal education, unemployment and single mother as risk factors for Ethiopian children [29]. Apart from socio-economic conditions, infant HIV exposure has been linked to certain biological factors that might influence growth: for example, chronic low grade immune activation [30], thymic dysfunction and lower levels of specific antibodies and CD4 cells [31], as well as higher risk of exposure to other pathogens. In addition, HIV-EU infants are also exposed to antiretroviral drugs, and feeding practices frequently differ to those recommended for HIV-unexposed children with shorter duration of breastfeeding. Recent studies indicate that infant exposure to HIV without perinatal infection is associated with increased infant mortality [11,12] and morbidity [10,13] but variable effects on growth [10,13,14].

Reports on growth of HIV-EU children show discordant results, which may partly be explained by different study designs and interventions for HIV-EU children. While inferior growth patterns have been reported in several studies, these studies differ from our design in that all not HIV-EU participated in PMTCT care until the time of growth measurement [16,32,33] and/or that weaning was considerably earlier in HIV-EU [32,33]. In agreement with our findings, other studies with continued PMTCT-like follow-up [34,35] and similar feeding practice [34] found non-inferior growth in HIV-EU infants.

Given the differences in design and settings, the reasons for the heterogeneous results must be interpreted with caution, but it is likely that participation in PMTCT follow-up care and access to a range of interventions could counteract several of the negative consequences on growth directly or indirectly associated with HIV exposure. Since we included HIV-U controls from the same uptake area we could exclude better socio-economic status among HIV-EU children in our study population as an explanation for these findings; on the contrary, HIV-EU children had higher prevalence of poverty indicators (single mother, poor level of education in the mother, only having one room in the home, and lack of access to running water). We were unable to assess a high number of infants born to HIV-positive women due to loss to follow-up of the infant and/or the mother. It is probable that these infants had worse outcomes (with regard to survival, morbidity and growth) than infants that remained in PMTCT follow-up and care at 9–12 months of age. Therefore, our conclusions of similar growth as HIV-U infants only apply to children with continuous attendance in PMTCT interventions. Yet, we believe that our findings emphasize the importance of health and nutrition interventions targeting especially vulnerable groups of children. Our data support that HIV-EU children constitute such a group; however, other groups of vulnerable children could also be identified. The fact that almost half of HIV-EU infants had been lost to follow-up before the age of 9–12 months also demonstrates the need to improve the performance of the Ethiopian PMTCT program.

Lack of maternal education was strongly associated with lower weight and length gain and stunting and wasting, consistent with previous reports [29,36,37]. In our population, the mode of infant feeding was not significantly associated with growth pattern. This is in contrast to other studies that have found inferior growth in children who were not breastfed. Although the Ethiopian guidelines recommend cessation of breastfeeding at the age of 6 months for children born to HIV-positive women, 75% of HIV-EU in our study were still breastfed at 9–12 months. Consequently, the non-breastfed population was small; furthermore, it is likely that early cessation of breastfeeding was mainly practised in wealthier families, a phenomenon that may have introduced a certain bias.

Several of the interventions included in PMTCT could be of benefit for the growth evolution of HIV-EU children. In particular, regular growth monitoring may detect growth deviations at early stages and allow for access to nutritional support. Recently, cotrimoxazole prophylaxis was shown to be linked to improved weight and height development in HIV-infected Asian infants with relatively preserved immune systems [38]. Although this intervention has not yet been investigated in HIV-EU children, it is possible that cotrimoxazole could also have a protective effect in this population (for example, for reduction of malaria and common bacterial infections), and this intervention is currently being studied in a randomized controlled trial in South Africa [39]. In addition, it is possible that receipt of full ART during pregnancy is associated with better infant growth evolution through improvements in maternal immune function.

Except for immunizations and deworming, the majority of Ethiopian infants are not routinely subject to follow-up with regards to growth. While nutritional support is provided free of charge once malnutrition is diagnosed, this relies on passive case finding. Our findings suggest that active case finding for infants with growth deviations in association with routine measles vaccination at 9–12 months of age might be feasible in the Ethiopian health system. In our cohort, this led to identification of underweight in 6.7% and stunting in 20.5% of 358 HIV-unexposed children.

To our knowledge, our study is the first to explore growth patterns in HIV-EU from Ethiopia, the second most populated country in sub-Saharan Africa with a large population of HIV-positive women of fertile age. We selected HIV-unexposed controls from the same uptake area, and collected data on a range of socio-economic variables of importance for child development that may act as confounders for associations between HIV exposure and growth.

### Limitations

In order to include an adequate number of HIV-EU children we identified these subjects retrospectively from PMTCT registers. Retrospective data were not available for HIV-U infants, hence we recruited these separately at a later time-point, using similar questionnaires and investigation procedures. Since this procedure may have entailed differences in data collection, our findings must be interpreted with some caution. Furthermore, our population of HIV-EU infants only includes subjects who remained in PMTCT care at 9–12 months of age, and for whom maternal data were available. It is also possible that HIV-U controls who present for measles vaccination represent a healthier subset of Ethiopian children; however, vaccination coverage in the uptake area is high (83% in 2013 [40]). Although misclassification with regard to HIV status may have occurred in some HIV-EU infants (with HIV seroconversion occurring after initial testing at 6 weeks of age), as well as among the controls (since maternal HIV serostatus was based on self-report), this effect would be minimal and unlikely to have an impact on our findings. Paradoxically, lack of access to running water was associated with lower risk for stunting in multivariate analysis. The reason for this association is not obvious, but might possibly reflect the presence of confounding factors for which data were missing. Furthermore, factors not included in our analysis could impact growth in HIV-EU children, for example maternal immune status and the presence of co-infections, in particular tuberculosis.

### Conclusions

We found non-inferior growth development at 9–12 months of age in HIV-EU infants compared to HIV-U controls from the same uptake area in Ethiopia, with higher WAZ among HIV-EU children, despite worse socio-economic status in families of HIV-EU infants. Growth retardation was associated with socio-demographic factors. In summary, our findings support a positive impact on infant growth in children remaining in PMTCT care, and suggest that children in the community at risk of growth retardation could be targeted for nutritional interventions through active case finding in association with routine measles vaccination.

## Paper context

HIV-EU constitute a vulnerable group, due to exposure to biological effects of maternal HIV infection, and, poorer socio-economic conditions over-represented in this group. Previous studies on their growth pattern differ in design and findings. Here, we find that Etiopoian HIV-EU infants adhering to PMTCT have non-inferior growth pattern despite worse socio-economic conditions. This emphasizes the importance of increasing the adherence to PMTCT for HIV-EU, and, possibly, benefits of growth monitoring for all Ethiopian infants.

## References

[1] Collins S. Treating severe acute malnutrition seriously. Arch Dis Child. 2007 ;92(5):453–10. 17449529 10.1136/adc.2006.098327 PMC2083726

[2] Kar BR, Rao SL, Chandramouli BA Chandramouli B a. Cognitive development in children with chronic protein energy malnutrition. Behav Brain Funct. 2008 1;4:31. 10.1186/1744-9081-4-31 PMC2519065 18652660

[3] Dewey KG, Begum K Long-term consequences of stunting in early life. Matern Child Nutr. 2011;7(Suppl 3):5–18. 21929633 10.1111/j.1740-8709.2011.00349.x PMC6860846

[4] Onis M WHO Child Growth Standards based on length/height, weight and age. Acta Paediatr. 2006:76-85. 10.1111/j.1651-2227.2006.tb02378.x 16817681

[5] Unicef, World Health Organization, The World Bank Joint Child Malnutrition Estimates: levels trends in child malnutrition. 2012 Available from: http://www.who.int/nutgrowthdb/estimates2011/en/

[6] Egata G, Berhane Y, Worku A Predictors of acute undernutrition among children aged 6 to 36 months in east rural Ethiopia: a community based nested case - control study. BMC Pediatr. 2014;14(1):91. 24708711 10.1186/1471-2431-14-91 PMC3994202

[7] Asfaw M, Wondaferash M, Taha M, et al Prevalence of undernutrition and associated factors among children aged between six to fifty nine months in Bule Hora district, South Ethiopia. BMC Public Health. 2015;15(1):41. 25636688 10.1186/s12889-015-1370-9 PMC4314803

[8] Krishna A, Oh J, Lee J, et al Short-term and long-term associations between household wealth and physical growth: a cross-comparative analysis of children from four low- and middle-income countries. Glob Health Action. 2015;8:26523. 10.3402/gha.v8.26523 PMC4320209 25660535

[9] Wamalwa D, Benki-Nugent S, Langat A, et al Survival benefit of early infant antiretroviral therapy is compromised when diagnosis is delayed. Pediatr Infect Dis J. 2012;31(7):729–731. 22544051 10.1097/INF.0b013e3182587796 PMC3756892

[10] The FS HIV-exposed, uninfected African child. Trop Med Int Health. 2013 3;14(3):276–287. 10.1111/j.1365-3156.2009.02220.x 19171011

[11] Marinda E, Humphrey JH, Iliff PJ, et al Child mortality according to maternal and infant HIV status in Zimbabwe. Pediatr Infect Dis J. 2007;26(6):519–526. 17529870 10.1097/01.inf.0000264527.69954.4c

[12] Landes M, van Lettow M, Chan AK, et al Mortality and health outcomes of HIV-exposed and unexposed children in a PMTCT cohort in Malawi. PLoS One. 2012 ;7(10):e47337. 10.1371/journal.pone.0047337 PMC3474798 23082157

[13] Le Roux S, Abrams EJ, Nguyen K, et al Clinical outcomes of HIV-exposed, -uninfected children in sub-Saharan Africa. Trop Med Int Health 2016;1–17. 10.1111/tmi.12716 27125333

[14] Evans C, Jones CE, Prendergast AJ HIV-exposed, uninfected infants: new global challenges in the era of paediatric HIV elimination. Lancet Infect Dis. 2016;16(6):e92–107. 27049574 10.1016/S1473-3099(16)00055-4

[15] Sherry B, Embree JE, Mei Z, et al Sociodemographic characteristics, care, feeding practices, and growth of cohorts of children born to HIV-1 seropositive and seronegative mothers in Nairobi, Kenya. Trop Med Int Health 2000;5(10): 678-686. 10.1046/j.1365-3156.2000.00631.x 11044261

[16] Muhangi L, Lule S, Mpairwe H, et al Maternal HIV infection and other factors associated with growth outcomes of HIV-uninfected infants in Entebbe, Uganda. Public Health Nutr. 2013;16:1548–1557. 23507372 10.1017/S1368980013000499 PMC3733066

[17] International CSA [Ethiopia] and I. Ethiopia Demographic and Health Survey 2011 [Internet]. Addis Ababa: Central Statistical Agency and ICF International; 2012 Available from: https://www.unicef.org/ethiopia/

[18] World Health Organization ECO. UPDATE |ETHIOPIA HIV /AIDS Progress in 2014 2015 Available from: http://www.afro.who.int/en/ethiopia/who-country-office-ethiopia.html

[19] HIV/AIDS Prevention and Control Office of Federal Democratic Republic of Ethiopia AIDS in Ethiopia. Sixth report. Natl AIDS Resour Cent Addis Ababa, Ethiop. 2006;368(9553):2119 Available from: http://www.etharc.org/aidsineth/publications/aidsineth6th_en.pdf

[20] Ethiopian Federal Ministry of Health. Guidelines For Prevention of Mother-to-Child Transmission of HIV In Ethiopia Federal HIV/AIDS Prevention and Control Office. Ethiopia: Addis Ababa, 2007.

[21] World Health Organisation WHO Anthro (version 3.2.2, January 2011) and Macros”. 2011 Available from: http://www.who.int/childgrowth/software/en/

[22] Kikafunda JK, Walker AF, Collett D, et al Risk factors for early childhood malnutrition in Uganda. Pediatrics [Internet]. 1998;102(4):e45–e45. 10.1542/peds.102.4.e45 9755282

[23] McDonald C, Kupka R, Manji K Predictors of stunting, wasting and underweight among Tanzanian children born to HIV-infected women. Eur J Clin Nutr. 2012;66(11):1265–1276. 23031850 10.1038/ejcn.2012.136 PMC3491141

[24] Jeyaseelan L, Lakshman M Risk factors for malnutrition in south Indian children. J Biosoc Sci. 1997;29(1):93–100. 9881122 10.1017/s002193209700093x

[25] Dasgupta P, Bhattacherjee S, Das DK Food security in households of people living with human immunodeficiency virus/acquired immunodeficiency syndrome: a cross-sectional study in a subdivision of Darjeeling district, West Bengal. J Prev Med Public Heal. 2016;49(4):240–248. 10.3961/jpmph.16.023 PMC4977769 27499166

[[26] Bukusuba J, Kikafunda JK, Whitehead RG Food security status in households of people living with HIV/AIDS (PLWHA) in a Ugandan urban setting. Br J Nutr. 2007;98(1):211–217. 17381879 10.1017/S0007114507691806

[27] Bairagi R On validity of some anthropometric indicators as predictors of mortality. [Internet]. Am J Clin Nutr. 1981;34:2592–2594. 6975565 10.1093/ajcn/34.11.2592

[28] Caulfield L, De Onis M, Blossner M, et al Undernutrition as an underlying cause of childs death associated with diarrhea, pneumonia, malaria and measles. Am J Clin Nutr. 2004;80(1):193–198. 15213048 10.1093/ajcn/80.1.193

[29] Girma W, Genebo T Determinants of the nutritional status of mothers and children in Ethiopia. Calverton (MD): USA ORC Macro; 2002.

[30] Schramm DB, Kuhn L, Gray GE, et al In vivo effects of HIV-1 exposure in the presence and absence of single-dose nevirapine on cellular plasma activation markers of infants born to HIV-1-seropositive mothers. J Acquir Immune Defic Syndr. 2006;42(5):545–553. 16837862 10.1097/01.qai.0000225009.30698.ce PMC2367220

[31] Nielsen SD, Jeppesen DL, Kolte L, et al Impaired progenitor cell function in HIV-negative infants of HIV-positive mothers results in decreased thymic output and low CD4 counts. Blood. 2001;98(2):398–404. 11435309 10.1182/blood.v98.2.398

[32] Filteau S, Baisley K, Chisenga M, et al Provision of micronutrient-fortified food from 6 months of age does not permit HIV-exposed uninfected Zambian children to catch up in growth to HIV-unexposed children: a randomized controlled trial. J Acquir Immune Defic Syndr. 2011;56(2):166–175. 21119523 10.1097/QAI.0b013e318201f6c9

[33] Moraleda C, de Deus N, Serna-Bolea C, et al Impact of HIV exposure on health outcomes in HIV-negative infants born to HIV-positive mothers in Sub-Saharan Africa. J Acquir Immune Defic Syndr. 2014;65(2):182–189. 24442224 10.1097/QAI.0000000000000019

[34] Patel D, Bland R, Coovadia H, et al Breastfeeding, HIV status and weights in South African children: a comparison of HIV-exposed and unexposed children. AIDS. 2010;24(3):437–445. 19915445 10.1097/QAD.0b013e3283345f91

[35] Ramokolo V, Lombard C, Fadnes LT, et al HIV infection, viral load, low birth weight, and nevirapine are independent influences on growth velocity in HIV-exposed South African infants. J Nutr. 2014;144(1):42–48. 24198309 10.3945/jn.113.178616

[36] Yimer G Malnutrition among children in Southern Ethiopia : levels and risk factors. Ethiop J Heal Dev. 2000;14(3):283–292.

[37] Astale T, Chenault M, Cormier SA Help-seeking behavior for children with Acute respiratory infection in Ethiopia: results from 2011 Ethiopia Demographic and Health Survey. PLoS One. 2015;10(11):1–10. 10.1371/journal.pone.0142553 PMC4641632 26560469

[38] Boettiger DC, Muktiarti D, Kurniati N, et al Early height and weight changes in children using cotrimoxazole prophylaxis with antiretroviral therapy. Clin Infect Dis. 2016;63:1236–1244. 27470239 10.1093/cid/ciw514 PMC5064162

[39] Coutsoudis A, Daniels B, Moodley-Govender E, et al Randomised controlled trial testing the effect of cotrimoxazole prophylaxis on morbidity and mortality outcomes in breastfed HIV-exposed uninfected infants: study protocol. BMJ Open [Internet]. 2016;6(7):e010656. 10.1136/bmjopen-2015-010656 PMC4947798 27406638

[40] Ethiopia |immunization - WHO |regional Office for Africa [Internet]. [cited 2016 10 30]. Available from: http://www.afro.who.int/en/ethiopia/country-programmes/topics/4594-ethiopia-immunization.html

